# Genetic Biomarkers in Melanoma of the Ocular Region: What the Medical Oncologist Should Know

**DOI:** 10.3390/ijms21155231

**Published:** 2020-07-23

**Authors:** Kalijn Fredrike Bol, Marco Donia, Steffen Heegaard, Jens Folke Kiilgaard, Inge Marie Svane

**Affiliations:** 1 National Center for Cancer Immune Therapy, Department of Oncology, Herlev Hospital, Copenhagen University Hospital, 2730 Herlev, Denmark; Kalijn.Fredrike.Bol@regionh.dk (K.F.B.); Marco.Donia@regionh.dk (M.D.); 2 Department of Ophthalmology, Rigshospitalet, Copenhagen University Hospital, 2100 Copenhagen, Denmark; Steffen.Heegaard@regionh.dk (S.H.); Jens.Folke.Kiilgaard@regionh.dk (J.F.K.); 3 Department of Pathology, Rigshospitalet, Copenhagen University Hospital, 2100 Copenhagen, Denmark

**Keywords:** posterior uveal melanoma, iris melanoma, conjunctival melanoma, ocular melanoma, genetic biomarkers, *BRAF* mutation

## Abstract

Melanoma of the ocular region (ocular melanoma) comprises about 5% of all patients with melanoma and covers posterior uveal melanoma, iris melanoma, and conjunctival melanoma. The risk of metastasis is much higher in patients with ocular melanoma compared to a primary melanoma of the skin. The subtypes of ocular melanoma have distinct genetic features, which should be taken into consideration when making clinical decisions. Most relevant for current practice is the absence of *BRAF* mutations in posterior uveal melanoma, although present in some iris melanomas and conjunctival melanomas. In this review, we discuss the genetic biomarkers of the subtypes of ocular melanoma and their impacts on the clinical care of these patients.

## 1. Introduction

The discovery of drugs targeting the mitogen-activated protein kinase (MAPK) pathway constitutes a major advancement in the treatment of patients with metastatic cutaneous melanoma harboring a somatic mutation in the *BRAF* gene on chromosome 7. Combined BRAF/MEK inhibition induces objective responses in approximately 65% of patients with a *BRAF* V600 mutation and improves the progression-free and overall survival [1,2,3]. Together with the emergence of immune checkpoint inhibitors [4,5], the prospects of patients with metastatic cutaneous melanoma are highly improved. However, not all these advancements can be applied directly to patients with non-cutaneous melanoma, i.e., of ocular or mucosal origin. 

**Figure 1:**
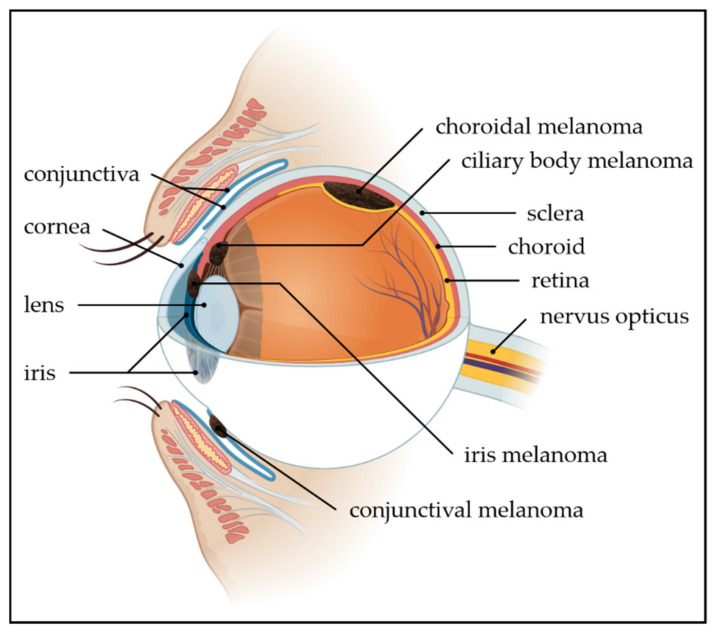
Anatomic location of melanomas of the ocular region. Melanomas of the ocular region arise from melanocytes in the conjunctiva (5%) or the uvea (95%). Of the uveal melanomas, approximately 90% arise in the choroid, 5% in the ciliary body, and 5% in the iris. (Figure was created with PowerPoint and BioRender.com).

In about 5% of all patients with melanoma, the primary tumor is of ocular origin (Figure 1). The majority of ocular melanomas (OcM) arise intraocularly, in the uvea. Uveal melanoma (UM) can be further divided into posterior UM (arising in the choroid or ciliary body) and iris melanoma. The risk to develop metastasis for patients with OcM is much higher compared to patients with a primary cutaneous melanoma and can be more than 50% in high-risk tumors of the posterior uvea [6,7,8]. The incidence rate of UM is 2–8 per million person/year, and most patients are diagnosed between the ages of 55 and 75 years [6,9]. 

A small subset of all OcM, approximately 5%, arise on the front of the eye from melanocytes in the basal layer of the epithelium of the conjunctival membrane [10,11]. Conjunctival melanomas arise de novo or from precursor lesions, such as nevi and primary-acquired melanosis with atypia [12,13]. As the conjunctiva is a mucous membrane, conjunctival melanomas are also classified as a subtype of mucosal melanoma. The incidence rate is ~0.5 per million person/year in Caucasians and peaks around the age of 60 years [12,14]. 

Although all originating in the ocular region, posterior UM, iris melanoma, and conjunctival melanoma have distinct genetic features and should be regarded as different disease subsets. Here, we will discuss the basics and genetic biomarkers of all subtypes of OcM for the practicing medical oncologist, with a clear focus on mutations that can serve as diagnostic, prognostic, or predictive biomarkers in the clinic.

## 2. Genetic Biomarkers at Initial Diagnosis of the Primary Tumor

### 2.1. Posterior Uveal Melanoma

UM originates in the choroid in ~90% of cases, ~5% in the ciliary body, and ~5% is confined to the iris [11,15]. Patients with posterior UM usually present with visual symptoms such as blurred vision, flashes of light, floaters, and/or visual field loss. Approximately 30% of patients are asymptomatic and are diagnosed upon routine examination [16]. UM is often a clinical diagnosis, without pathological assessment, based on examination by an ophthalmologist specialized in ocular oncology. Local management depends, among other factors, on the tumor size and location. Treatment can consist of eye-sparing treatment, e.g., (plaque or proton) radiotherapy or enucleation. 

Posterior UM is staged according to the American Joint Committee on Cancer (AJCC) staging system for UM, in which both posterior UM and iris melanoma are incorporated but with a distinct primary tumor (T) classification. The tumor size, based on both the basal diameter and thickness, is the predominant clinical predictor of prognosis in primary posterior UM [17]. The T category is further defined by the involvement of the ciliary body together with extraocular extension. As a lymphatic drainage system is absent for intraocular structures, lymphatic spread can only occur when extraocular growth is present and is seldomly seen. As a consequence, the stage groups are different compared to cutaneous melanoma. In UM, the stages I to IIIC all refer to primary tumors without metastasis, and stage IV defines both regional (N1) and distant metastasis (M1). About a third of patients have stage I disease, a bit more than half of patients have stage II disease, and approximately one in ten patients have stage III disease at diagnosis. Only about 2% of patients show detectable metastasis at initial diagnosis [17]. The five-year survival rate of primary posterior UM decreases from 96% in stage I disease to 26% in stage IIIC disease. Although genetic biomarkers are not incorporated in the current AJCC staging system, they endorse the importance of genetic profiling. It is shown that the addition of genetic biomarkers can enhance the prognostication of survival [18]. 

Most posterior UM harbor a driver mutation in *GNAQ* (~55%) or *GNA11* (~40%) (Table 1) [19]. Mutations in *GNAQ* and *GNA11* are mutually exclusive and can lead to the activation of multiple downstream pathways involved in proliferation and cell growth [20]. As both mutations are already observed in most uveal nevi [21], they are not suitable as a diagnostic biomarker and neither have they been shown to be of significant prognostic value, although *GNA11*-mutated tumors might be slightly more aggressive than their *GNAQ*-mutated counterparts [22,23]. In contrast, some chromosomal aberrations and secondary driver mutations are strong prognostic genetic biomarkers. Of the chromosomal aberrations, loss of chromosome 3 with or without gains of chromosome 8q is associated with a high risk of metastatic disease (>50%) and occurs in approximately half of the patients. Disomy 3 and a gain in chromosome 6p are low-risk features and, when present, rarely lead to metastasis [24]. Two online prediction models, PRiMeUM and LUMPO, and a visual nomogram include chromosomal aberrations to predict the risk of metastasis and mortality, respectively [25,26,27].

The most important secondary driver mutations occur in *BAP1*, *SF3B1*, or *EIF1AX* and are generally mutually exclusive. They are present in ~45%, ~25%, and ~20% of the primary posterior UM, respectively [19]. *BRAF* and *NRAS* mutations, frequently occurring in cutaneous melanoma, do not occur in posterior UM [28]. *KIT* mutations are rare [28,29,30]. Inactivation of the tumor-suppressor gene *BAP1*, located on chromosome 3, usually occurs by a *BAP1* mutation combined with monosomy 3. *BAP1* germline mutations are only present in ~2% of patients with UM but should be considered in the presence of a family history of UM, cutaneous melanoma, mesothelioma, or renal cell carcinoma [31]. *BAP1* inactivation gives a high risk of metastatic disease [32]. Mutations in the splicing gene *SF3B1*, located on chromosome 2, occur mainly in disomy 3 tumors and give an intermediate risk to developing metastasis, occurring late compared to *BAP1*-mutated tumors [33]. UM with mutations in *EIF1AX*, located on the X chromosome, are usually also only present in disomy 3 tumors and seldomly metastasize [19]. Although exceptional, *BAP1* mutations can occur in combination with disomy 3 and *SF3B1* or *EIF1AX* mutations in combination with monosomy 3. In addition, albeit often described as mutually exclusive mutations, *SF3B1* mutations are described in combination with *EIF1AX* or *BAP1* mutations [30,34].

**Figure 2:**
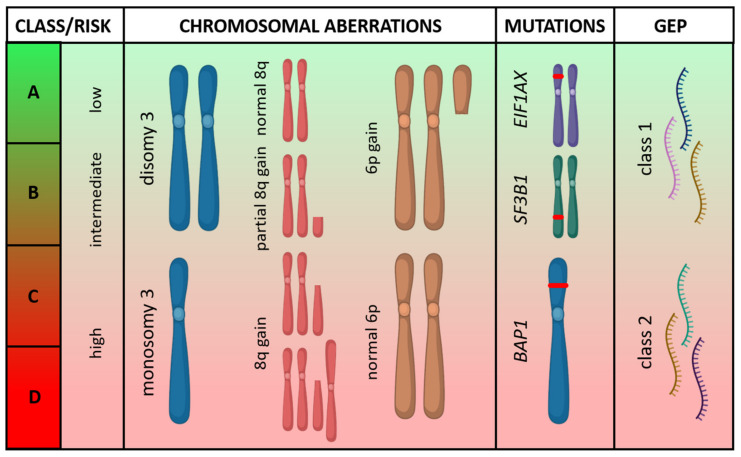
Overview of genetic prognostic biomarkers in primary posterior uveal melanoma. Based on their increasing risk of metastasis, four molecular subsets (**A**–**D**) are depicted based on their chromosomal aberrations, secondary driver mutations, and gene expression profile (GEP). (Figure was created with PowerPoint and BioRender.com).

Another option to predict the risk of metastasis is gene expression profiling (GEP) of 15 genes. GEP differentiates class 1 (low-risk) and class 2 (high-risk) tumors, with a five-year risk of metastasis of ~4% for GEP class 1 and ~51% for GEP class 2 tumors [35,36]. GEP class 1 tumors mainly contain tumors with disomy 3 and *EIF1AX* or *SF3B1* mutations, where monosomy 3 tumors with *BAP1* mutations are mainly classified as GEP class 2 tumors. Recently, a combined classification was proposed describing four molecular subsets of posterior UM (Figure 2) [28,30,37]. 

In cutaneous melanoma, high mutation frequencies are attributed to the mutagenic effect of UV radiation. A UV-induced mutational signature, with a high fraction of C>T transitions, is present in about 80% of cutaneous melanomas [38]. As the cornea and lens absorb almost all UV radiation, UV-induced mutational signatures are absent in posterior UM. The mutational load is among the lowest of all cancer types, comparable to that of pediatric cancers [28,30]. 

To look into the prognostic biomarkers, tumor material is required for analyses. Preferably, fresh tumor material is used for genetic or transcriptomic analyses or formalin-fixed paraffin-embedded material for *BAP1* inactivation testing, which can be determined by protein expression immunohistochemistry. In patients undergoing enucleation of the eye, tissue will be available. However, the majority of patients are currently treated with an eye-conserving strategy. In these patients, the option to perform a tumor biopsy to obtain tumor tissue for the testing of prognostic biomarkers should be discussed prior to treatment. Tumor biopsies in posterior UM are considered a safe procedure and do not increase the risk of metastasis when performed by an experienced ocular surgeon [39]. 

Following local treatment, patients are monitored for local recurrence and the development of metastasis. UM spreads almost solely hematogenously, as extraocular growth is present in less than 2% of patients and lymphatic spread cannot occur from within the eye [17]. With rarely any regional lymph node metastasis, sentinel lymph node biopsies are not performed. Recommendations for follow-up vary widely and may depend on the presence of high-risk features but usually include at least a full ophthalmological examination and imaging of the liver every 3–12 months up to 10 years. With half-yearly screening of the liver by MRI, metastases were detected before symptoms in 92% of patients [40]. In patients with known low-risk features, regular imaging of the liver may be omitted.

### 2.2. Iris Melanoma

Only ~5% of all UM are confined to the iris. Iris melanomas have a separate definition of the T categories in the AJCC staging system for UM based on the tumor size, tumor extension, and presence of secondary glaucoma. No prognostic stage groups are defined. As the iris color changes and a tumor can be noticed by the patient, iris melanoma is often diagnosed at an earlier stage compared to posterior UM. Almost all patients are diagnosed with a T1 or T2 primary tumor, and less than 5% of patients have a T3 or T4 tumor. The five-year survival rate of iris melanoma decreases from 100% in patients with T1 tumors to 50% in T4 tumors. Overall, metastasis occurs in ~5–10% of patients—only in the presence of extension into the ciliary body, choroid, or sclera—and the prognosis is relatively good [11,41,42,43]. The standard treatment for iris melanoma can consist of active observation (watchful waiting), tumor resection, or proton beam radiation. 

In iris melanoma, genetic profiling data is limited, as the disease is rare, and little material is available, as lesions are often small. The available data shows that a loss of chromosome 3 and the same mutations as in posterior UM can be found, although their prognostic value is uncertain. In contrast to posterior UM, mutations in *BRAF* (0–47%) and *NRAS* have been described in iris melanoma, although the frequency of their presence is unclear, and they might not represent driver mutations (Table 1) [44,45,46,47]. As the iris is not protected from UV damage, UV-induced mutational signatures have recently been detected in iris melanoma [48,49].

### 2.3. Conjunctival Melanoma

Patients with conjunctival melanoma typically present with a pigmented lesion on the bulbar conjunctiva. Conjunctival melanomas of the non-bulbar conjunctiva and non-pigmented lesions are less common [13]. Slit-lamp examination can establish the clinical diagnosis, and diagnostic incisional biopsies should be avoided due to the increased risk of recurrence [50]. Surgical excision of the primary tumor is the standard treatment, often supplemented with local adjuvant treatment to lower the risk of recurrence. Local adjuvant treatment can consist of cryotherapy, topical chemotherapy, topical immunotherapy, plaque brachytherapy, or proton beam radiotherapy. Of the various available local adjuvant therapies, cryotherapy is utilized most often. 

Conjunctival melanoma has its own AJCC staging system, which contains no subdivisions if regional lymph node metastases (N1) or distant metastases (M1) are present and no prognostic stage groups are defined. T categories, based on size and location, correlate clearly with risk of metastasis and survival, with five-year survival rates decreasing from 97% (T1) to 58% (T3; with survival rates for T4 unknown). Only 5% of patients are diagnosed with a T3 primary tumor, and T4 tumors are seldomly seen [51]. In addition to the AJCC T category, ulceration of the primary tumor and sentinel node involvement are of prognostic value [51,52]. Despite local adjuvant treatment, the risk of local recurrence is high (30–60%) [13,51]. Nodal involvement occurs in ~15% of patients, either detected at diagnosis or during follow-up. A sentinel lymph node biopsy can be considered if the primary tumor is thicker than 2 mm and can be expected to be positive in ~5% of patients—up to 20% in selected patients [51,52]. Finally, about 20–30% of patients develop distant metastasis [13,52,53]. Detectable metastasis at the initial diagnosis are rare (2%) [51]. Guidelines on the follow-up of patients are lacking, but the high risks of local recurrence and systemic spread support regular ophthalmological examinations and imaging of the regional lymph nodes and internal organs (e.g., ultrasound of cervical lymph nodes andor (PET/)CT of neck, thorax, and the abdomen). 

In contrast to posterior UM and other mucosal melanoma subtypes, conjunctival melanoma quite frequently expresses *BRAF* mutations (~20–55% of patients; Table 1) [54,55,56,57]. *NRAS* is mutated in ~20% of conjunctival melanomas, and *KIT* mutations are reported in 0–7% [29,56,58]. Unfortunately, none of the mutations seem to be of prognostic relevance [56,57]. In-line with cutaneous melanoma, UV radiation plays a role in the development of conjunctival melanoma; UV-induced mutation signatures are demonstrated, as well as a high mutational load [59].

## 3. Genetic Biomarkers When Metastatic Disease Is Present

### 3.1. Posterior Uveal Melanoma

Posterior UM may metastasize years before treatment of the primary tumor [63,64]. Still, in only 2% of patients, dissemination is detected at diagnosis, even when screened with PET/CT [65]. Most patients that develop metastatic disease are diagnosed with metastasis within five years after initial diagnosis, but detection may occur decades later [7]. The most common site of metastasis is the liver, which is affected in 90% of patients and the sole site of metastasis in ~50% of patients. Less common sites, often presenting later during the disease course, include the lungs, bones, subcutaneous tissues, and lymph nodes. Brain metastases are uncommon (~5%) and do not occur as the primary metastatic site [66,67]. It is recommended to confirm metastases by histopathology. The M category of the AJCC staging system is different from cutaneous melanoma and solely based on the diameter of the largest metastasis (M1a ≤ 3 m, M1b 3.1–8.0 cm, and M1c ≥ 8.1 cm), which strongly correlates with survival [68]. Other factors correlating with survival, similar to cutaneous melanoma, are the performance status and lactate dehydrogenase (LDH) level [69]. 

The genetic high-risk features, such as monosomy 3 and BAP1 mutations, are more frequently seen in patients with metastatic disease [23]. Whether any of the genetic alterations are also of prognostic value once metastases are present is unknown and, thus, abates the reason for genetic testing if not determined at first presentation. This includes the analyses of mutations in *GNAQ* and *GNA11*, which were hoped to be predictive of MEK inhibition, as the mutations constitutively activate the MAPK pathway. Despite promising results in a phase II trial, a phase III clinical trial with MEK inhibition combined with chemotherapy in patients with metastatic UM did not show benefits over chemotherapy. The objective response rate was 3% in the arm with MEK inhibition plus chemotherapy and 0% in the arm with chemotherapy (dacarbazine) alone [70]. *KIT* mutations rarely occur in posterior UM, and UM patients were not included in the phase II clinical trials studying the effect of the tyrosine kinase inhibitor imatinib in *KIT*-mutated melanoma. The response rates in these trials were moderate and may be limited to patients with *KIT* mutations in certain hotspots of clinical relevance [71,72,73]. The very low mutation frequency and the lack of documented responses to KIT inhibition in posterior UM makes mutation analyses and the off-label use of imatinib in UM debatable. 

For patients with metastatic UM, a standard of care is lacking. Surgical resection or other liver-directed therapies, including hepatic perfusion with chemotherapy, may result in long-term survival, but data is sparse, highly biased, and the procedures are only feasible in highly selected patients [74,75]. Systemic treatments with chemotherapy or immunotherapy, as approved for metastatic cutaneous melanoma, are often considered. No meaningful benefit has been shown with chemotherapeutic regimens. In-line with the low mutational load of posterior UM [28], the use of immune checkpoint inhibitors has shown limited responses compared to cutaneous melanoma [75,76,77], though moderate evidence from real-world data suggests some efficacy of combined treatment with anti-PD1 and anti-CTLA4 antibodies [78,79]. Results of a phase II clinical trial with the combination as any line of treatment and as the first-line treatment are awaited. Other forms of immunotherapy, including adoptive T cell transfer, adjuvant dendritic cell vaccination, and a bispecific molecule targeting T cells and gp100 (tebentafusp), are tested in clinical trials. The latter gained a fast-track designation by the FDA for HLA-A * 0201-positive metastatic UM patients. In two phase I clinical trials, partial responses were observed in 14–18% of patients. Data are awaited from a phase II clinical trial, and inclusion is ongoing in a second phase II clinical trial [80]. No randomization takes place in any of these phase II trials with immunotherapy, so comparisons with other treatments will be difficult, but response rates might be a good indicator of efficacy, as responses are extremely rare with chemotherapy.

The median overall survival of patients with metastatic UM is approximately four months in unselected patients and around 10 months in selected patients included in clinical trials [69,74]. As responses to treatment in the metastatic setting are scarce, the adjuvant treatment of high-risk UM patients, besides in clinical trials, is not recommended. Trial participation is limited due to the low availability of UM-specific trials and the exclusion of patients with UM from larger (cutaneous) melanoma clinical trials. In summary, the prognosis of metastatic UM is poor, and treatments with proven benefits are currently lacking. 

### 3.2. Iris Melanoma

Metastatic iris melanoma is extremely rare due to the low incidence of primary iris melanoma and their low risk of metastatic spread. Therefore, experience with the treatment of metastatic iris melanoma is scarce, and little is documented. It could be hypothesized that immune checkpoint inhibitors have possible efficacy, as iris melanoma shows the same UV-induced mutational signatures as cutaneous melanoma [48,49]. As for targeted therapy, it is unknown whether *BRAF*-mutated iris melanoma responds to BRAF(/MEK) inhibition, as it is not described in the literature. As *BRAF* mutations do occur in iris melanoma, genetic testing to detect *BRAF* mutations can be considered. However, the mutations detected so far are not located at codon 600 and might represent passenger mutations instead of driver mutations [44,45,47], further complicating the assumptions about possible efficacy. Overall, evidence-based treatment is impossible, and available options should be discussed carefully with the patient.

### 3.3. Conjunctival Melanoma

Distant metastasis occur in 20–30% of patients with a primary conjunctival melanoma and are often preceded by regional lymph node metastasis [13,52,53]. Not unlike cutaneous melanoma, all organs may be affected by metastasis, with the liver and lungs being the most common sites. Brain metastases are present in ~10–20% of patients [51]. Due to the rarity of the disease, no conjunctival melanoma-specific clinical trials have been performed. Treatment decisions should be based on experiences in mucosal and cutaneous melanoma and case reports of patients with conjunctival melanoma.

Patients with *BRAF*-mutated metastatic conjunctival melanoma have shown objective responses to BRAF inhibition in several reported cases but not in all [81,82,83,84]. BRAF/MEK inhibition showed the stable disease in one metastatic patient and (near) complete responses in two patients with local recurrent disease [85,86,87]. Although response rates cannot be determined and publication bias is likely present, responses to BRAF(/MEK) inhibition clearly occur, e.g., in contrast to *BRAF*-mutated colon cancer [88], and should be considered for patients with *BRAF*-mutated metastatic conjunctival melanoma. Patients with metastatic mucosal melanoma were included in the phase II trials with imatinib in *KIT*-mutated melanoma, but conjunctival melanomas are not separately mentioned [71,72,73]. As with UM, the off-label use of imatinib should be considered with care in patients with *KIT*-mutated conjunctival melanoma, as little is known about the chance of success. A better alternative might be the treatment with immune checkpoint inhibitors, which showed response rates of 23% with anti-PD1 treatment and 37% with combined anti-PD1/anti-CTLA4 treatment in patients with mucosal melanoma [89]. The number of patients with conjunctival melanoma in this cohort is not documented. However, case reports showing responses to immune checkpoint inhibition, including responses to anti-CTLA4 monotherapy, anti-PD1 monotherapy, and combined anti-PD1/anti-CTLA4 therapy, support this treatment option in conjunctival melanoma [90,91]. This is in-line with the expectations based on the UV signature and high mutational load seen in conjunctival melanoma [59]. Data on the survival of patients with metastatic conjunctival melanoma is limited but seems poor, with a median overall survival of 5–8 months, although survival is likely to have improved after BRAF(/MEK) inhibitors and immune checkpoint inhibitors became available [53,92]. As responses to both treatment modalities might (almost) be as good as in cutaneous melanoma, adjuvant treatment could be considered, although evidence is lacking. Additionally, neo-adjuvant BRAF/MEK inhibition is worth consideration in irresectable primary or local recurrent tumors with a *BRAF* mutation [86,93]. 

### 3.4. Melanoma of Unknown Primary

Approximately 2% of patients with melanoma present with regional or distant metastasis as the first manifestation of the disease without a known primary melanoma [10]. These patients represent about 10–15% of all patients with hematogenously spread melanoma at any timepoint during their disease course [94,95]. Immunohistochemical markers used for diagnostic purposes of melanoma, e.g., HMB45 (gp100), tyrosinase, and Melan-A, are also often expressed in OcM and, thus, not able to distinguish between the different melanoma subtypes. 

Melanoma of unknown primary is assumed to be arisen from a primary melanoma that spontaneously regressed. Random screening for a primary tumor by ophthalmoscopy, laryngoscopy, endoscopy of the lower gastrointestinal tract, and a gynecological examination is not recommended, as they rarely reveal a primary tumor [96]. The mutation profile of melanoma of unknown primary is similar to cutaneous melanoma, with frequent *BRAF* (~50%) and *NRAS* (~20%) mutations [97]. The detection of *KIT*, *GNA11*, or *GNAQ* mutations might warrant further screening for a primary mucosal or primary UM, as *GNAQ/GNA11* mutations are generally mutually exclusive with *BRAF/NRAS* mutations. Still, exceptions exist, and one of the mutations may represent a passenger mutation. For example, one of our patients with widespread metastases of melanoma showed both a *GNA11* Q209L and a *BRAF* V600K mutation. Ophthalmological examination, skin examination, and imaging did not reveal a primary tumor, and no objective response was obtained with BRAF/MEK inhibition. Overall, patients with melanoma of unknown primary should be treated as cutaneous melanoma patients and have a similar prognosis [10,95,98].

## 4. Conclusions

Strong prognostic genetic biomarkers predicting the development of metastasis, including chromosomal aberrations, DNA mutations, and RNA profiles, are available in primary posterior UM. In patients planned for eye-conserving local treatment, the option to perform a tumor biopsy to obtain tumor tissue for genetic testing should be discussed prior to treatment. The genetic biomarkers improve the accuracy of predicting the individual prognosis, may influence the schedule of follow-up, and might allow for adjuvant treatments in clinical trials. Currently, no standard (adjuvant) treatment is available for patients with high-risk or metastatic UM. When patients with UM develop metastasis and visit the department of Medical Oncology, the strong prognostic biomarkers are not known to be of further clinical relevance. In addition, *BRAF* mutations are absent in posterior UM, and testing for the *BRAF* status in this patient group is ineffectual. The *BRAF* status in patients with metastatic iris melanoma might be the only relevant predictive biomarker, although responsiveness to BRAF(/MEK) inhibition is unknown. For all patients with metastatic UM, clinical trial participation is recommended—however, rarely accessible. Of the available treatments, the combined treatment with anti-PD1/anti-CTLA4 therapy is likely to be the best treatment option at present, at least until the bispecific biologic tebentafusp reaches the market. 

In conjunctival melanoma, little is known on prognostic genetic biomarkers due to the low incidence of the disease, albeit mutations are frequently present. However, in the metastatic setting, BRAF(/MEK) inhibition can induce clinical responses, and, thus, the *BRAF* status is a valuable predictive genetic biomarker. Besides immunotherapy, treatment with BRAF(/MEK) inhibitors should be considered in metastatic *BRAF*-mutated conjunctival melanoma and can even be considered in the (neo)adjuvant setting. Successful targeting of other mutations might be possible in the future, both in uveal and conjunctival melanoma, but the rarity of the diseases causes research to move forward slowly.

## Figures and Tables

**Table 1:** Driver mutations in subtypes of primary melanoma.

Gene Mutation	*BRAF*	*NRAS*	*KIT*	*GNA11*	*GNAQ*	*BAP1*	*SF3B1*	*EIF1AX*
Posterior uveal melanoma	-	-	2%	40%	55%	45%	25%	20%
Iris melanoma	15%	5%	5%	25%	55%	25%	4%	25%
Conjunctival melanoma	35%	20%	5%	-	-	-	-	-
Cutaneous melanoma *	40%	20%	<5%	-	-	<1%	-	-

Percentages of gene mutations in primary tumors are estimates based on the literature. The mutations *BRAF* and *NRAS*; *GNA11* and *GNAQ*; and *BAP1*, *SF3B1*, and *EIF1AX* are generally mutually exclusive. - = Mutation frequency is close to 0%. * = References: [60,61,62].

## Abbreviations

AJCC	American Joint Committee on Cancer
GEP	Gene expression profiling
LDH	Lactate dehydrogenase
MAPK	Mitogen-activated protein kinase
OcM	Ocular melanoma
UM	Uveal melanoma

## References

[1] Robert C., Karaszewska B., Schachter J., Rutkowski P., Mackiewicz A., Stroiakovski D., Lichinitser M., Dummer R., Grange F., Mortier L. (2015). Improved overall survival in melanoma with combined dabrafenib and trametinib. N. Engl. J. Med..

[2] Ascierto P.A., McArthur G.A., Dreno B., Atkinson V., Liszkay G., Di Giacomo A.M., Mandala M., Demidov L., Stroyakovskiy D., Thomas L. (2016). Cobimetinib combined with vemurafenib in advanced BRAF(V600)-mutant melanoma (coBRIM): Updated efficacy results from a randomised, double-blind, phase 3 trial. Lancet Oncol..

[3] Dummer R., Ascierto P.A., Gogas H.J., Arance A., Mandala M., Liszkay G., Garbe C., Schadendorf D., Krajsova I., Gutzmer R. (2018). Encorafenib plus binimetinib versus vemurafenib or encorafenib in patients with BRAF-mutant melanoma (COLUMBUS): A multicentre, open-label, randomised phase 3 trial. Lancet Oncol..

[4] Larkin J., Chiarion-Sileni V., Gonzalez R., Grob J.J., Cowey C.L., Lao C.D., Schadendorf D., Dummer R., Smylie M., Rutkowski P. (2015). Combined Nivolumab and Ipilimumab or Monotherapy in Untreated Melanoma. N. Engl. J. Med..

[5] Robert C., Schachter J., Long G.V., Arance A., Grob J.J., Mortier L., Daud A., Carlino M.S., McNeil C., Lotem M. (2015). Pembrolizumab versus Ipilimumab in Advanced Melanoma. N. Engl. J. Med..

[6] Singh A.D., Turell M.E., Topham A.K. (2011). Uveal melanoma: Trends in incidence, treatment, and survival. Ophthalmology.

[7] Kujala E., Makitie T., Kivela T. (2003). Very long-term prognosis of patients with malignant uveal melanoma. Invest. Ophthalmol. Vis. Sci..

[8] Jensen O.A. (1982). Malignant melanomas of the human uvea: 25-year follow-up of cases in Denmark, 1943–1952. Acta Ophthalmol..

[9] Virgili G., Gatta G., Ciccolallo L., Capocaccia R., Biggeri A., Crocetti E., Lutz J.M., Paci E., Group E.W. (2007). Incidence of uveal melanoma in Europe. Ophthalmology.

[10] Chang A.E., Karnell L.H., Menck H.R. (1998). The National Cancer Data Base report on cutaneous and noncutaneous melanoma: A summary of 84,836 cases from the past decade. The American College of Surgeons Commission on Cancer and the American Cancer Society. Cancer.

[11] Isager P., Osterlind A., Engholm G., Heegaard S., Lindegaard J., Overgaard J., Storm H.H. (2005). Uveal and conjunctival malignant melanoma in Denmark, 1943–1997: Incidence and validation study. Ophthalmic. Epidemiol..

[12] Norregaard J.C., Gerner N., Jensen O.A., Prause J.U. (1996). Malignant melanoma of the conjunctiva: Occurrence and survival following surgery and radiotherapy in a Danish population. Graefes Arch. Clin. Exp. Ophthalmol..

[13] Shields C.L., Shields J.A., Gunduz K., Cater J., Mercado G.V., Gross N., Lally B. (2000). Conjunctival melanoma: Risk factors for recurrence, exenteration, metastasis, and death in 150 consecutive patients. Arch. Ophthalmol..

[14] McLaughlin C.C., Wu X.C., Jemal A., Martin H.J., Roche L.M., Chen V.W. (2005). Incidence of noncutaneous melanomas in the U.S. Cancer.

[15] Shields C.L., Furuta M., Thangappan A., Nagori S., Mashayekhi A., Lally D.R., Kelly C.C., Rudich D.S., Nagori A.V., Wakade O.A. (2009). Metastasis of uveal melanoma millimeter-by-millimeter in 8033 consecutive eyes. Arch. Ophthalmol..

[16] Damato E.M., Damato B.E. (2012). Detection and time to treatment of uveal melanoma in the United Kingdom: An evaluation of 2,384 patients. Ophthalmology.

[17] AJCC Ophthalmic Oncology Task Force (2015). International Validation of the American Joint Committee on Cancer’s 7th Edition Classification of Uveal Melanoma. JAMA Ophthalmol..

[18] Dogrusoz M., Bagger M., van Duinen S.G., Kroes W.G., Ruivenkamp C.A., Bohringer S., Andersen K.K., Luyten G.P., Kiilgaard J.F., Jager M.J. (2017). The Prognostic Value of AJCC Staging in Uveal Melanoma Is Enhanced by Adding Chromosome 3 and 8q Status. Invest. Ophthalmol. Vis. Sci..

[19] Smit K.N., Jager M.J., de Klein A., Kili E. (2020). Uveal melanoma: Towards a molecular understanding. Prog. Retin. Eye Res..

[20] Chua V., Lapadula D., Randolph C., Benovic J.L., Wedegaertner P.B., Aplin A.E. (2017). Dysregulated GPCR Signaling and Therapeutic Options in Uveal Melanoma. Mol. Cancer Res..

[21] Vader M.J.C., Madigan M.C., Versluis M., Suleiman H.M., Gezgin G., Gruis N.A., Out-Luiting J.J., Bergman W., Verdijk R.M., Jager M.J. (2017). GNAQ and GNA11 mutations and downstream YAP activation in choroidal nevi. Br. J. Cancer.

[22] Koopmans A.E., Vaarwater J., Paridaens D., Naus N.C., Kilic E., de Klein A., Rotterdam Ocular Melanoma Study Group (2013). Patient survival in uveal melanoma is not affected by oncogenic mutations in GNAQ and GNA11. Br. J. Cancer.

[23] Griewank K.G., van de Nes J., Schilling B., Moll I., Sucker A., Kakavand H., Haydu L.E., Asher M., Zimmer L., Hillen U. (2014). Genetic and clinico-pathologic analysis of metastatic uveal melanoma. Mod. Pathol..

[24] Damato B., Eleuteri A., Taktak A.F., Coupland S.E. (2011). Estimating prognosis for survival after treatment of choroidal melanoma. Prog. Retin. Eye Res..

[25] DeParis S.W., Taktak A., Eleuteri A., Enanoria W., Heimann H., Coupland S.E., Damato B. (2016). External Validation of the Liverpool Uveal Melanoma Prognosticator Online. Invest. Ophthalmol. Vis. Sci..

[26] Vaquero-Garcia J., Lalonde E., Ewens K.G., Ebrahimzadeh J., Richard-Yutz J., Shields C.L., Barrera A., Green C.J., Barash Y., Ganguly A. (2017). PRiMeUM: A Model for Predicting Risk of Metastasis in Uveal Melanoma. Invest. Ophthalmol. Vis. Sci..

[27] Bagger M.M. (2018). Intraocular biopsy of uveal melanoma Risk assessment and identification of genetic prognostic markers. Acta. Ophthalmol..

[28] Royer-Bertrand B., Torsello M., Rimoldi D., El Zaoui I., Cisarova K., Pescini-Gobert R., Raynaud F., Zografos L., Schalenbourg A., Speiser D. (2016). Comprehensive Genetic Landscape of Uveal Melanoma by Whole-Genome Sequencing. Am. J. Hum. Genet..

[29] Wallander M.L., Layfield L.J., Emerson L.L., Mamalis N., Davis D., Tripp S.R., Holden J.A. (2011). KIT mutations in ocular melanoma: Frequency and anatomic distribution. Mod. Pathol..

[30] Robertson A.G., Shih J., Yau C., Gibb E.A., Oba J., Mungall K.L., Hess J.M., Uzunangelov V., Walter V., Danilova L. (2017). Integrative Analysis Identifies Four Molecular and Clinical Subsets in Uveal Melanoma. Cancer Cell.

[31] Gupta M.P., Lane A.M., DeAngelis M.M., Mayne K., Crabtree M., Gragoudas E.S., Kim I.K. (2015). Clinical Characteristics of Uveal Melanoma in Patients With Germline BAP1 Mutations. JAMA Ophthalmol..

[32] Harbour J.W., Onken M.D., Roberson E.D., Duan S., Cao L., Worley L.A., Council M.L., Matatall K.A., Helms C., Bowcock A.M. (2010). Frequent mutation of BAP1 in metastasizing uveal melanomas. Science.

[33] Yavuzyigitoglu S., Koopmans A.E., Verdijk R.M., Vaarwater J., Eussen B., van Bodegom A., Paridaens D., Kilic E., de Klein A., Rotterdam Ocular Melanoma Study Group (2016). Uveal Melanomas with SF3B1 Mutations: A Distinct Subclass Associated with Late-Onset Metastases. Ophthalmology.

[34] Thornton S., Coupland S.E., Olohan L., Sibbring J.S., Kenny J.G., Hertz-Fowler C., Liu X., Haldenby S., Heimann H., Hussain R. (2020). Targeted Next-Generation Sequencing of 117 Routine Clinical Samples Provides Further Insights into the Molecular Landscape of Uveal Melanoma. Cancers.

[35] Onken M.D., Worley L.A., Char D.H., Augsburger J.J., Correa Z.M., Nudleman E., Aaberg T.M., Altaweel M.M., Bardenstein D.S., Finger P.T. (2012). Collaborative Ocular Oncology Group report number 1: Prospective validation of a multi-gene prognostic assay in uveal melanoma. Ophthalmology.

[36] Binkley E.M., Bena J.F., Davanzo J.M., Hinz C., Boldt H.C., Singh A.D. (2020). Gene Expression Profiling Prognostication of Posterior Uveal Melanoma: Does Size Matter?. Ophthalmol. Retin..

[37] Jager M.J., Brouwer N.J., Esmaeli B. (2018). The Cancer Genome Atlas Project: An Integrated Molecular View of Uveal Melanoma. Ophthalmology.

[38] Cancer Genome Atlas Network (2015). Genomic Classification of Cutaneous Melanoma. Cell.

[39] Bagger M., Smidt-Nielsen I., Andersen M.K., Jensen P.K., Heegaard S., Andersen K.K., Friis S., Kiilgaard J.F. (2018). Long-Term Metastatic Risk after Biopsy of Posterior Uveal Melanoma. Ophthalmology.

[40] Marshall E., Romaniuk C., Ghaneh P., Wong H., McKay M., Chopra M., Coupland S.E., Damato B.E. (2013). MRI in the detection of hepatic metastases from high-risk uveal melanoma: A prospective study in 188 patients. Br. J. Ophthalmol..

[41] Shields C.L., Kaliki S., Shah S.U., Luo W., Furuta M., Shields J.A. (2012). Iris melanoma: Features and prognosis in 317 children and adults. J. AAPOS.

[42] Khan S., Finger P.T., Yu G.P., Razzaq L., Jager M.J., de Keizer R.J., Sandkull P., Seregard S., Gologorsky D., Schefler A.C. (2012). Clinical and pathologic characteristics of biopsy-proven iris melanoma: A multicenter international study. Arch. Ophthalmol..

[43] Isager P., Engholm G., Overgaard J., Storm H. (2006). Uveal and conjunctival malignant melanoma in denmark 1943-97: Observed and relative survival of patients followed through 2002. Ophthalmic. Epidemiol..

[44] Van Poppelen N.M., Vaarwater J., Mudhar H.S., Sisley K., Rennie I.G., Rundle P., Brands T., van den Bosch Q.C.C., Mensink H.W., de Klein A. (2018). Genetic Background of Iris Melanomas and Iris Melanocytic Tumors of Uncertain Malignant Potential. Ophthalmology.

[45] Henriquez F., Janssen C., Kemp E.G., Roberts F. (2007). The T1799A BRAF mutation is present in iris melanoma. Invest. Ophthalmol. Vis. Sci..

[46] Scholz S.L., Moller I., Reis H., Susskind D., van de Nes J.A.P., Leonardelli S., Schilling B., Livingstone E., Schimming T., Paschen A. (2017). Frequent GNAQ, GNA11, and EIF1AX Mutations in Iris Melanoma. Invest. Ophthalmol. Vis. Sci..

[47] Krishna Y., Kalirai H., Thornton S., Damato B.E., Heimann H., Coupland S.E. (2016). Genetic findings in treatment-naive and proton-beam-radiated iris melanomas. Br. J. Ophthalmol..

[48] Karlsson J., Nilsson L.M., Mitra S., Alsen S., Shelke G.V., Sah V.R., Forsberg E.M.V., Stierner U., All-Eriksson C., Einarsdottir B. (2020). Molecular profiling of driver events in metastatic uveal melanoma. Nat. Commun..

[49] Johansson P.A., Brooks K., Newell F., Palmer J.M., Wilmott J.S., Pritchard A.L., Broit N., Wood S., Carlino M.S., Leonard C. (2020). Whole genome landscapes of uveal melanoma show an ultraviolet radiation signature in iris tumours. Nat. Commun..

[50] Larsen A.C., Dahmcke C.M., Dahl C., Siersma V.D., Toft P.B., Coupland S.E., Prause J.U., Guldberg P., Heegaard S. (2015). A Retrospective Review of Conjunctival Melanoma Presentation, Treatment, and Outcome and an Investigation of Features Associated With BRAF Mutations. JAMA Ophthalmol..

[51] Jain P., Finger P.T., Damato B., Coupland S.E., Heimann H., Kenawy N., Brouwer N.J., Marinkovic M., Van Duinen S.G., Caujolle J.P. (2019). Multicenter, International Assessment of the Eighth Edition of the American Joint Committee on Cancer Cancer Staging Manual for Conjunctival Melanoma. JAMA Ophthalmol..

[52] Esmaeli B., Rubin M.L., Xu S., Goepfert R.P., Curry J.L., Prieto V.G., Ning J., Tetzlaff M.T. (2019). Greater Tumor Thickness, Ulceration, and Positive Sentinel Lymph Node Are Associated With Worse Prognosis in Patients With Conjunctival Melanoma: Implications for Future AJCC Classifications. Am. J. Surg. Pathol..

[53] Missotten G.S., Keijser S., De Keizer R.J., De Wolff-Rouendaal D. (2005). Conjunctival melanoma in the Netherlands: A nationwide study. Invest. Ophthalmol. Vis. Sci..

[54] Gear H., Williams H., Kemp E.G., Roberts F. (2004). BRAF mutations in conjunctival melanoma. Invest. Ophthalmol. Vis. Sci..

[55] Lake S.L., Jmor F., Dopierala J., Taktak A.F., Coupland S.E., Damato B.E. (2011). Multiplex ligation-dependent probe amplification of conjunctival melanoma reveals common BRAF V600E gene mutation and gene copy number changes. Invest. Ophthalmol. Vis. Sci..

[56] Griewank K.G., Westekemper H., Murali R., Mach M., Schilling B., Wiesner T., Schimming T., Livingstone E., Sucker A., Grabellus F. (2013). Conjunctival melanomas harbor BRAF and NRAS mutations and copy number changes similar to cutaneous and mucosal melanomas. Clin. Cancer Res..

[57] Larsen A.C., Dahl C., Dahmcke C.M., Lade-Keller J., Siersma V.D., Toft P.B., Coupland S.E., Prause J.U., Guldberg P., Heegaard S. (2016). BRAF mutations in conjunctival melanoma: Investigation of incidence, clinicopathological features, prognosis and paired premalignant lesions. Acta. Ophthalmol..

[58] Beadling C., Jacobson-Dunlop E., Hodi F.S., Le C., Warrick A., Patterson J., Town A., Harlow A., Cruz F., Azar S. (2008). KIT gene mutations and copy number in melanoma subtypes. Clin. Cancer Res..

[59] Rivolta C., Royer-Bertrand B., Rimoldi D., Schalenbourg A., Zografos L., Leyvraz S., Moulin A. (2016). UV light signature in conjunctival melanoma; not only skin should be protected from solar radiation. J. Hum. Genet..

[60] Platz A., Egyhazi S., Ringborg U., Hansson J. (2008). Human cutaneous melanoma; a review of NRAS and BRAF mutation frequencies in relation to histogenetic subclass and body site. Mol. Oncol..

[61] Hayward N.K., Wilmott J.S., Waddell N., Johansson P.A., Field M.A., Nones K., Patch A.M., Kakavand H., Alexandrov L.B., Burke H. (2017). Whole-genome landscapes of major melanoma subtypes. Nature.

[62] O’Shea S.J., Robles-Espinoza C.D., McLellan L., Harrigan J., Jacq X., Hewinson J., Iyer V., Merchant W., Elliott F., Harland M. (2017). A population-based analysis of germline BAP1 mutations in melanoma. Hum. Mol. Genet..

[63] Eskelin S., Pyrhonen S., Summanen P., Hahka-Kemppinen M., Kivela T. (2000). Tumor doubling times in metastatic malignant melanoma of the uvea: Tumor progression before and after treatment. Ophthalmology.

[64] Shain A.H., Bagger M.M., Yu R., Chang D., Liu S., Vemula S., Weier J.F., Wadt K., Heegaard S., Bastian B.C. (2019). The genetic evolution of metastatic uveal melanoma. Nat. Genet..

[65] Freton A., Chin K.J., Raut R., Tena L.B., Kivela T., Finger P.T. (2012). Initial PET/CT staging for choroidal melanoma: AJCC correlation and second nonocular primaries in 333 patients. Eur. J. Ophthalmol..

[66] Diener-West M., Reynolds S.M., Agugliaro D.J., Caldwell R., Cumming K., Earle J.D., Hawkins B.S., Hayman J.A., Jaiyesimi I., Jampol L.M. (2005). Development of metastatic disease after enrollment in the COMS trials for treatment of choroidal melanoma: Collaborative Ocular Melanoma Study Group Report No. 26. Arch. Ophthalmol..

[67] Lorigan J.G., Wallace S., Mavligit G.M. (1991). The prevalence and location of metastases from ocular melanoma: Imaging study in 110 patients. AJR. Am. J. Roentgenol..

[68] Amin M.B., Edge S.B., Greene F.L., Schilsky R.L., Gaspar L.E., Washington M.K., Sullivan D.C., Brookland R.K., Brierley J.D., Balch C.M. (2017). AJCC Cancer Staging Man.

[69] Khoja L., Atenafu E.G., Suciu S., Leyvraz S., Sato T., Marshall E., Keilholz U., Zimmer L., Patel S.P., Piperno-Neumann S. (2019). Meta-analysis in metastatic uveal melanoma to determine progression free and overall survival benchmarks: An international rare cancers initiative (IRCI) ocular melanoma study. Ann. Oncol..

[70] Carvajal R.D., Piperno-Neumann S., Kapiteijn E., Chapman P.B., Frank S., Joshua A.M., Piulats J.M., Wolter P., Cocquyt V., Chmielowski B. (2018). Selumetinib in Combination With Dacarbazine in Patients With Metastatic Uveal Melanoma: A Phase III, Multicenter, Randomized Trial (SUMIT). J. Clin. Oncol..

[71] Carvajal R.D., Antonescu C.R., Wolchok J.D., Chapman P.B., Roman R.A., Teitcher J., Panageas K.S., Busam K.J., Chmielowski B., Lutzky J. (2011). KIT as a therapeutic target in metastatic melanoma. JAMA.

[72] Hodi F.S., Corless C.L., Giobbie-Hurder A., Fletcher J.A., Zhu M., Marino-Enriquez A., Friedlander P., Gonzalez R., Weber J.S., Gajewski T.F. (2013). Imatinib for melanomas harboring mutationally activated or amplified KIT arising on mucosal, acral, and chronically sun-damaged skin. J. Clin. Oncol..

[73] Guo J., Si L., Kong Y., Flaherty K.T., Xu X., Zhu Y., Corless C.L., Li L., Li H., Sheng X. (2011). Phase II, open-label, single-arm trial of imatinib mesylate in patients with metastatic melanoma harboring c-Kit mutation or amplification. J. Clin. Oncol..

[74] Augsburger J.J., Correa Z.M., Shaikh A.H. (2009). Effectiveness of treatments for metastatic uveal melanoma. Am. J. Ophthalmol..

[75] Rantala E.S., Hernberg M., Kivela T.T. (2019). Overall survival after treatment for metastatic uveal melanoma: A systematic review and meta-analysis. Melanoma Res..

[76] Heppt M.V., Steeb T., Schlager J.G., Rosumeck S., Dressler C., Ruzicka T., Nast A., Berking C. (2017). Immune checkpoint blockade for unresectable or metastatic uveal melanoma: A systematic review. Cancer Treat. Rev..

[77] Yarchoan M., Hopkins A., Jaffee E.M. (2017). Tumor Mutational Burden and Response Rate to PD-1 Inhibition. N. Engl. J. Med..

[78] Bol K.F., Ellebaek E., Hoejberg L., Bagger M.M., Larsen M.S., Klausen T.W., Kohler U.H., Schmidt H., Bastholt L., Kiilgaard J.F. (2019). Real-World Impact of Immune Checkpoint Inhibitors in Metastatic Uveal Melanoma. Cancers.

[79] Heppt M.V., Amaral T., Kahler K.C., Heinzerling L., Hassel J.C., Meissner M., Kreuzberg N., Loquai C., Reinhardt L., Utikal J. (2019). Combined immune checkpoint blockade for metastatic uveal melanoma: A retrospective, multi-center study. J. Immunother. Cancer.

[80] Damato B.E., Dukes J., Goodall H., Carvajal R.D. (2019). Tebentafusp: T Cell Redirection for the Treatment of Metastatic Uveal Melanoma. Cancers.

[81] Griewank K.G., Westekemper H., Schilling B., Livingstone E., Schimming T., Sucker A., Hillen U., Steuhl K.P., Zimmer L., Schadendorf D. (2013). Conjunctival melanomas harbor BRAF and NRAS mutations--response. Clin. Cancer Res..

[82] Maleka A., Astrom G., Bystrom P., Ullenhag G.J. (2016). A case report of a patient with metastatic ocular melanoma who experienced a response to treatment with the BRAF inhibitor vemurafenib. BMC Cancer.

[83] Pinto Torres S., Andre T., Gouveia E., Costa L., Passos M.J. (2017). Systemic Treatment of Metastatic Conjunctival Melanoma. Case Rep. Oncol. Med..

[84] Weber J.L., Smalley K.S., Sondak V.K., Gibney G.T. (2013). Conjunctival melanomas harbor BRAF and NRAS mutations--Letter. Clin. Cancer Res..

[85] Rossi E., Maiorano B.A., Pagliara M.M., Sammarco M.G., Dosa T., Martini M., Rindi G., Bria E., Blasi M.A., Tortora G. (2019). Dabrafenib and Trametinib in BRAF Mutant Metastatic Conjunctival Melanoma. Front. Oncol..

[86] Dagi Glass L.R., Lawrence D.P., Jakobiec F.A., Freitag S.K. (2017). Conjunctival Melanoma Responsive to Combined Systemic BRAF/MEK Inhibitors. Ophthalmic Plast. Reconstr. Surg..

[87] Kim J.M., Weiss S., Sinard J.H., Pointdujour-Lim R. (2020). Dabrafenib and Trametinib for BRAF-Mutated Conjunctival Melanoma. Ocul. Oncol. Pathol..

[88] Kopetz S., Desai J., Chan E., Hecht J.R., O’Dwyer P.J., Maru D., Morris V., Janku F., Dasari A., Chung W. (2015). Phase II Pilot Study of Vemurafenib in Patients With Metastatic BRAF-Mutated Colorectal Cancer. J. Clin. Oncol..

[89] D’Angelo S.P., Larkin J., Sosman J.A., Lebbe C., Brady B., Neyns B., Schmidt H., Hassel J.C., Hodi F.S., Lorigan P. (2017). Efficacy and Safety of Nivolumab Alone or in Combination With Ipilimumab in Patients With Mucosal Melanoma: A Pooled Analysis. J. Clin. Oncol..

[90] Finger P.T., Pavlick A.C. (2019). Checkpoint inhibition immunotherapy for advanced local and systemic conjunctival melanoma: A clinical case series. J. Immunother. Cancer.

[91] Sagiv O., Thakar S.D., Kandl T.J., Ford J., Sniegowski M.C., Hwu W.J., Esmaeli B. (2018). Immunotherapy With Programmed Cell Death 1 Inhibitors for 5 Patients With Conjunctival Melanoma. JAMA Ophthalmol..

[92] Tuomaala S., Kivela T. (2004). Metastatic pattern and survival in disseminated conjunctival melanoma: Implications for sentinel lymph node biopsy. Ophthalmology.

[93] Pahlitzsch M., Bertelmann E., Mai C. (2014). Conjunctival melanoma and BRAF inhibitor therapy. Case Rep. Oncol. Med..

[94] Vallet A., Oriano B., Mortier L., Dalle S., Dutriaux C., Guillot B., Leccia M.T., Dalac S., Saiag P., Lacour J.P. (2019). Association of Time From Primary Diagnosis to First Distant Relapse of Metastatic Melanoma With Progression of Disease and Survival. JAMA Dermatol..

[95] Ellebaek E., Bastholt L., Schmidt H., Svane I.M., Donia M. (2019). The real-world outcome of metastatic melanoma: Unknown primary vs. known cutaneous. Int. J. Cancer.

[96] Tos T., Klyver H., Drzewiecki K.T. (2011). Extensive screening for primary tumor is redundant in melanoma of unknown primary. J. Surg. Oncol..

[97] Egberts F., Bergner I., Kruger S., Haag J., Behrens H.M., Hauschild A., Rocken C. (2014). Metastatic melanoma of unknown primary resembles the genotype of cutaneous melanomas. Ann. Oncol..

[98] Lee C.C., Faries M.B., Wanek L.A., Morton D.L. (2009). Improved survival for stage IV melanoma from an unknown primary site. J. Clin. Oncol..

